# Design and Implementation of Medical Ultrasound Remote Control Software Based on Sensor Design

**DOI:** 10.3389/fbioe.2020.620237

**Published:** 2021-02-11

**Authors:** Shuhao Deng, Quan Jiang, Yingchun Zhang, Xin Lu, Xiurong Shi, Yuan Zhang

**Affiliations:** ^1^Department of Ultrasound, Pudong New Area Peoples' Hospital Affiliated to Shanghai University of Medicine & Health Sciences, Shanghai, China; ^2^Second Affiliated Hospital of Soochow University, Suzhou, China; ^3^Shanghai Pudong New Area Public Interest Hospital, Pudong, China

**Keywords:** sensor, medical ultrasound, remote control, screen sharing, computer control

## Abstract

With the development of medical technology, medical ultrasound technology is widely used in the diagnosis of human diseases. It has become an indispensable diagnostic method in modern clinical medicine by detecting the internal physiology or tissue structure of the human body by ultrasound and then discovering diseases. Based on this, this paper designs a medical ultrasonic remote control software based on sensor design. The system software communicates with the stimulator through the Bluetooth port of the mobile phone and can send the parameter information input by the mobile phone to the field-programmable gate array of the stimulator for compilation. The upper computer control interface with a remote communication function is written by Lab VIEW software. A socket is used to establish inter-network process connection, and medical ultrasonic equipment simulates hardware key input according to the received control instructions so as to achieve the purpose of remote control. Experiments show that, compared with other systems, the infrared human body temperature measurement system with the functions of environmental temperature compensation and distance compensation can effectively reduce the influence of environmental temperature, distance, and other factors and has the advantages of non-contact, low power consumption, fast response speed, high sensitivity, and high accuracy, which is suitable for rapid and accurate human body temperature measurement in crowded places with large traffic.

## Introduction

At present, power ultrasound is widely used in machinery, electronics, electrical appliances, metallurgy, chemical industry, medicine, energy, materials, textiles, agriculture, environmental protection, and many other important fields (Xiaobin et al., 2017). Under this social background, mobile medicine, and telemedicine have gained wide application prospects. In the field of medical ultrasound, all kinds of ultrasonic examination equipment have the characteristics of large volume and inconvenient transportation, which makes clinicians only operate in front of ultrasonic equipment when performing an ultrasonic examination on patients, and has certain limitations (Yao et al., 2018; Wang et al., 2019). With the improvement of people's quality and quality of life, people pay more and more attention to their own health, hoping that potential diseases can be diagnosed and treated in advance. Using one of the most commonly used mobile terminals in life can meet the needs of doctors not only to control ultrasonic equipment but also to view real-time images displayed on the screen and manage remote files.

An ultrasonic sensor is developed by using the characteristics of ultrasonic (Li et al., 2017). In the past, ultrasonic detection mainly used manual detection, that is, according to the experience of professional inspectors, moving the detection probe to detect the object, so human factors have a great influence on the accuracy of detection. Moreover, this method has low detection efficiency, high cost, easy to cause false detection, and missed detection and is not suitable for remote automatic detection. In this paper, the network architecture of the ultrasonic remote application is designed to realize the remote control of the ultrasonic vibration system. By connecting the Internet with the server or controlling the ultrasonic diagnosis system through Bluetooth, the client interface is used to access the examinee's file and understand the examinee's information. The Ethernet communication between the multi-axis motion controller and the upper computer is realized by using User Datagram Protocol, and the remote automatic control of ultrasonic probe motion is realized, which improves the efficiency of ultrasonic detection and has important application value.

## Related Work

In China, ultrasonic diagnosis technology began in the 1960s. In recent years, technology has made rapid progress, and medical ultrasonic technology has also made great progress. Thanks to the rapid progress of human beings in expanding outer space in the middle of the last century, telemedicine has attracted more and more attention from medical practitioners. Hospitals, research institutions, and institutions of higher learning in Germany, Britain, Spain, Sweden, and the Netherlands have jointly put forward a plan called MobiHealth, which automatically collects the physiological parameters of patients by measuring and sensing equipment and uses wireless communication technology to transmit the obtained parameters to the monitoring center, so as to achieve the purpose of sharing medical resources and better rescuing patients. Burnik et al. (2019) developed the Otelo (Mobile Tele-Echo Geography Using an Ultra-Light Robot) system. This system is a mobile mechanical remote ultrasonic control system based on 4G point-to-point communication. The system uses the H.263 coded ultrasound image stream. It is verified that the image information can be transmitted in real-time in a 4G network environment. In recent years, to balance the distribution of medical resources, the state has promulgated targeted policies and invested a lot of money to establish service nodes in some central areas of provinces and promote the development of local telemedicine networks.

## Design Scheme

### System Architecture

**Figure 1:**
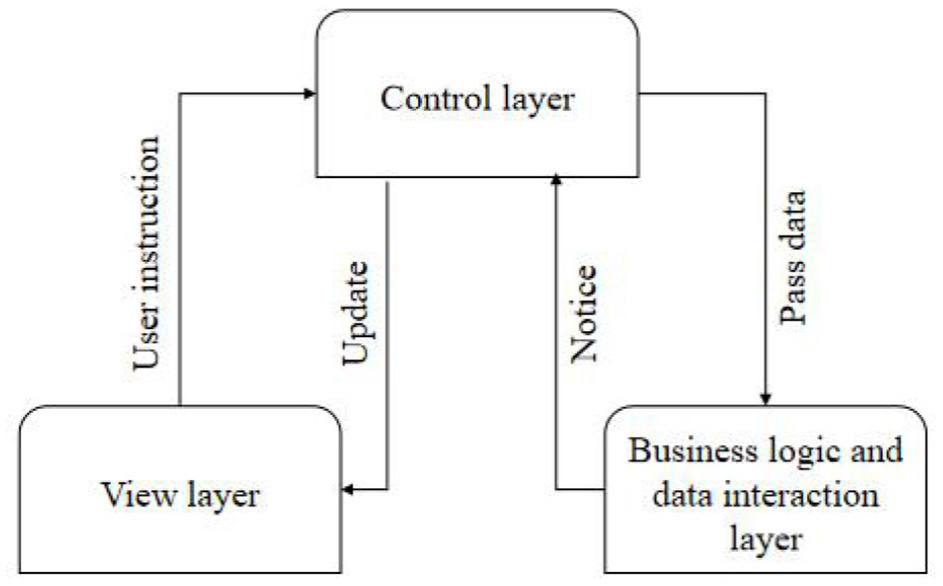
Interaction between layers of Model–View–Controller pattern.

This system needs two people to complete all the work: an ultrasonic imaging expert at the far end and an operator at the near end. Operators do not need much professional knowledge of ultrasonic imaging and only need to know the basic use of the instrument (Wei, 2018). This is because good software architecture can greatly reduce the cost of software development, improve the efficiency of software, and prolong the life cycle of software. As shown in Figure 1, it is the interactive relationship among all layers of the Model–View–Controller (MVC) pattern. The Controller is the bridge between View and Model, which ensures the synchronization between View and Model. After the Controller accepts the instruction from View and gives it to Model for logical processing, Model informs the Controller to update View according to the processing result (Yingjuan et al., 2017; Wang and Liying, 2018). The ultrasonic transducer chips at the transmitting end and the receiving end are packaged in a cylindrical shell. To ensure higher receiving efficiency at the receiving end, the centers of the transmitting surface and the receiving surface should be kept in a horizontal plane as far as possible. The upper computer sends a data request to the server (lower computer) as a client. When returning data, the upper computer reads the data returned by the lower computer as a server.

The whole system consists of client, ultrasonic equipment, and server program on the equipment. Each control cabinet contains 24 ultrasonic vibration systems, and each ultrasonic vibration system includes a numerical control ultrasonic generator and a 2-kW vibrator. The core of the monitoring terminal is the development board based on an ARM9 processor, which can be connected with the sensor modules for collecting physiological parameters such as blood pressure, respiration, blood oxygen, body temperature, and electrocardiogram (ECG). The main module of the monitoring center is the upper computer application software system on the computer, which can receive the measurement signals transmitted by the monitoring terminal and complete the functions of displaying, storing, and analyzing the measurement data. The system can be assembled by installing the corresponding software on the client and server computers (Chen et al., 2019). However, the system may not normally work at this time, so it is necessary to test the connectivity of the network and choose the way to establish the connection according to the network conditions.

### Design of Front-End Parameter Control and Display Module

In this module, the used probe needs to be automatically identified; the interface displays the probe information (probe name and picture) and the corresponding diagnosis type information and monitors whether the probe changes at any time and operates when the probe changes. The remote control module is the core of this system, which realizes that the remote computer can observe the real-time ultrasound images on the screen of the near-end computer through the remote desktop technology and can send control commands through the operation interface of the near-end computer. If the liquid level is lower than the detection surface of the sensor, the liquid density on the ultrasonic propagation path will decrease, the received signal energy will be attenuated, and the reflected waveform will decrease rapidly in amplitude with the decrease of the liquid level (Liangsheng and Chenglong, 2017). Because MVC is not suitable for small applications, strictly separating the view, controller, and model will make the code structure very complicated, and it is not easy to carry out subsequent development. Therefore, only separating the view layer from the data layer will not only make the classes displayed on the interface simpler but also make the code structure clearer.

**Figure 2:**
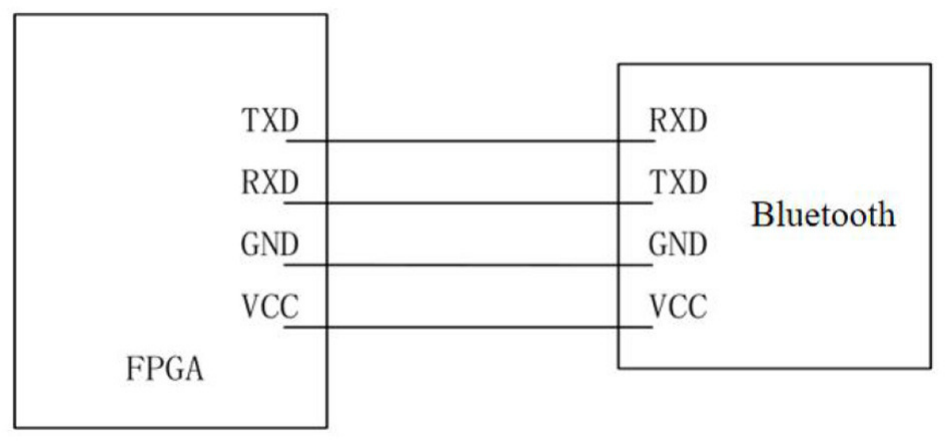
Communication diagram between FPGA and Bluetooth module.

The Bluetooth serial port module of the stimulator is located on the field-programmable gate array (FPGA) control board. The Bluetooth model used is HC-05, which is a high-performance Bluetooth serial port module and can be used for pairing with various intelligent terminals such as computers, Bluetooth hosts, and mobile phones with Bluetooth function. The baud rate range of this module is wide, ranging from 4,800 to 1,382,400 bits/s. The wiring mode of the communication circuit between the Bluetooth module and FPGA is shown in Figure 2, in which TX is the signal sending end, RX is the signal receiving end, GND is the ground, and the power supply VCC is 3.3 V.

**Figure 3:**
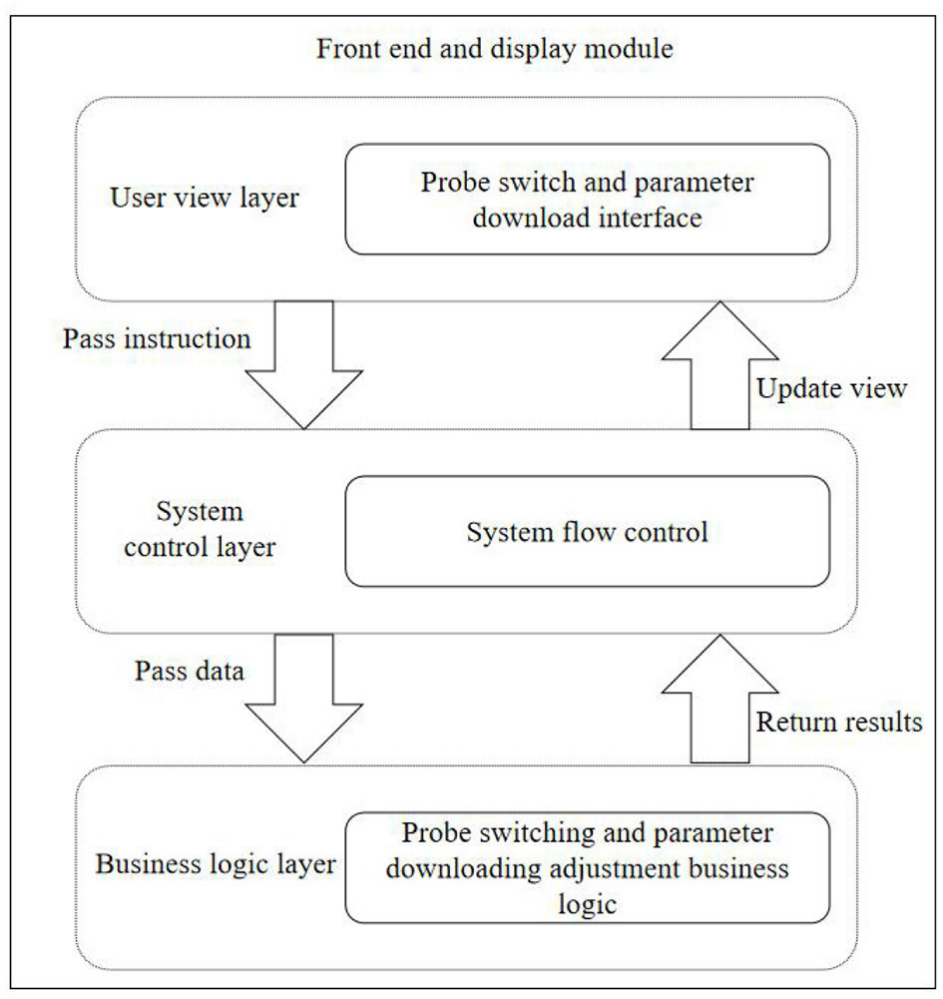
Front-end and display module hierarchy.

When selecting a diagnosis type, it is necessary to read the control parameters corresponding to the diagnosis type (Ruoyu et al., 2020). These control parameters are divided into front-end parameters and desired state configuration parameters. The front-end parameters are downloaded to the front-end to obtain different echo data for changing the image display quality. This sub-module also adopts the MVC mode, and the software structure level is shown in Figure 3.

A mechanical actuator is installed on the stepping motor to fix the ultrasonic probe, and a limit switch is installed on the mechanical actuator to control the motion range of the motor. Bluetooth communication is widely used as a wireless communication method with strong anti-interference ability, low cost, and low power consumption. During the measurement, the arterial blood flow is blocked by the cuff, and the pressure sensor is used to detect the oscillation wave of the gas in the cuff during the gradual deflation process (Shuli, 2018; Yanxia and Helin, 2020). The function of the remote controller is realized by establishing a Socket connection and simulating key input, that is, medical ultrasonic equipment and mobile terminal equipment are placed in the same local area network (LAN), and the internet protocol (IP) address of medical ultrasonic equipment is an input to establish a corresponding connection with a mobile terminal. Each ultrasonic generator shall have an automatic frequency searching function without a manual setting. At the same time, the system should ensure that the amplitude of each ultrasonic vibration system is constant and adapt to the change of workload.

### Software Design of Data Transmission Module

In this paper, the monitoring terminal of telemedicine system separately designed the storage function to realize the storage of five physiological parameters and related values, including heart rate value, blood oxygen value, pulse rate value, respiration value, blood pressure value (systolic pressure, diastolic pressure, and average pressure), and body temperature value in turn. Under this requirement, it is necessary to design an ultrasonic image remote sharing module, which can clearly display the ultrasonic images and related parameters on the ultrasonic equipment to the client of the mobile equipment. The data collected from the ultrasonic generator are forwarded to the touch screen and operator station by multicast communication. In addition, the instructions from the touch screen or operator station are received in a point-to-point manner, and the ultrasonic generator is controlled by the Modbus protocol. The function of control system software is designed on Linux operating platform, and the remote communication between the upper computer and motion controller is realized by the user datagram protocol (Hongping et al., 2017).

**Figure 4:**
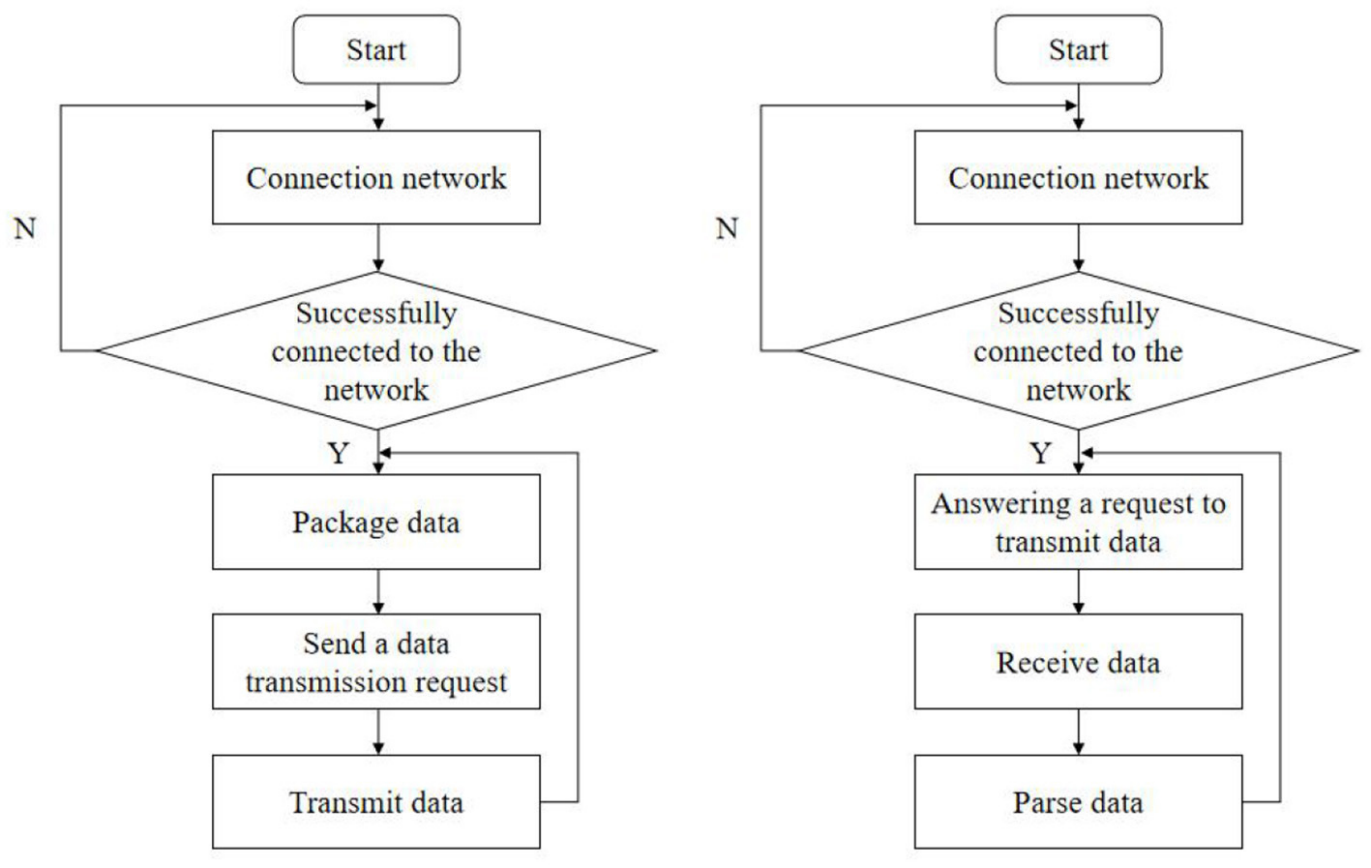
Flow chart of data transmission module.

The data transmission module of the monitoring terminal is divided into two parts: the data sending module and the data receiving module. Figure 4 is the program flow chart of the transmission module.

The ultrasonic transducer chips at the transmitting end and the receiving end are packaged in a cylindrical shell. To ensure higher receiving efficiency at the receiving end, the centers of the transmitting surface and the receiving surface should be kept on a horizontal plane as far as possible. The transport layer is located in the fourth layer of the seven-layer network model. The upper three layers process data and provide interfaces for users to operate conveniently, whereas the lower three layers transmit data, and the transport layer is located in the middle for isolation, encapsulating the communication interface of the lower layer and hiding the contents. It is mainly responsible for receiving the data transmitted from the user view layer and handing it over to the corresponding business logic class for processing. In this module, the system control layer is composed of Android Manifest.xml, monitoring events, and Activity jumps (Hongli et al., 2019).

The sensor module should communicate with the main controller in the system to receive commands such as start-up detection and resolution setting and send alarm signals to the host. Due to the limitation of the PICl2F675 pin and the requirement of long-distance data exchange, parallel data transmission cannot be realized. After the connection is established, the ARM processor encapsulates the collected physiological data according to the data protocol and sends a data transmission request to the upper computer monitoring software. After receiving the response, it sends a data packet to the upper computer monitoring software. At this time, the upper computer monitoring software analyzes the data packet and displays the data and waveform of physiological parameters. The data management module mainly realizes importing, viewing, and deleting various files stored in the database, displays them in the file list according to the time sequence of the year, month, and day of the files, views all the files on a certain date, queries the files according to keywords, and displays the contents of the searched files.

### Ultrasonic Transmitting and Receiving Circuit

**Figure 5:**
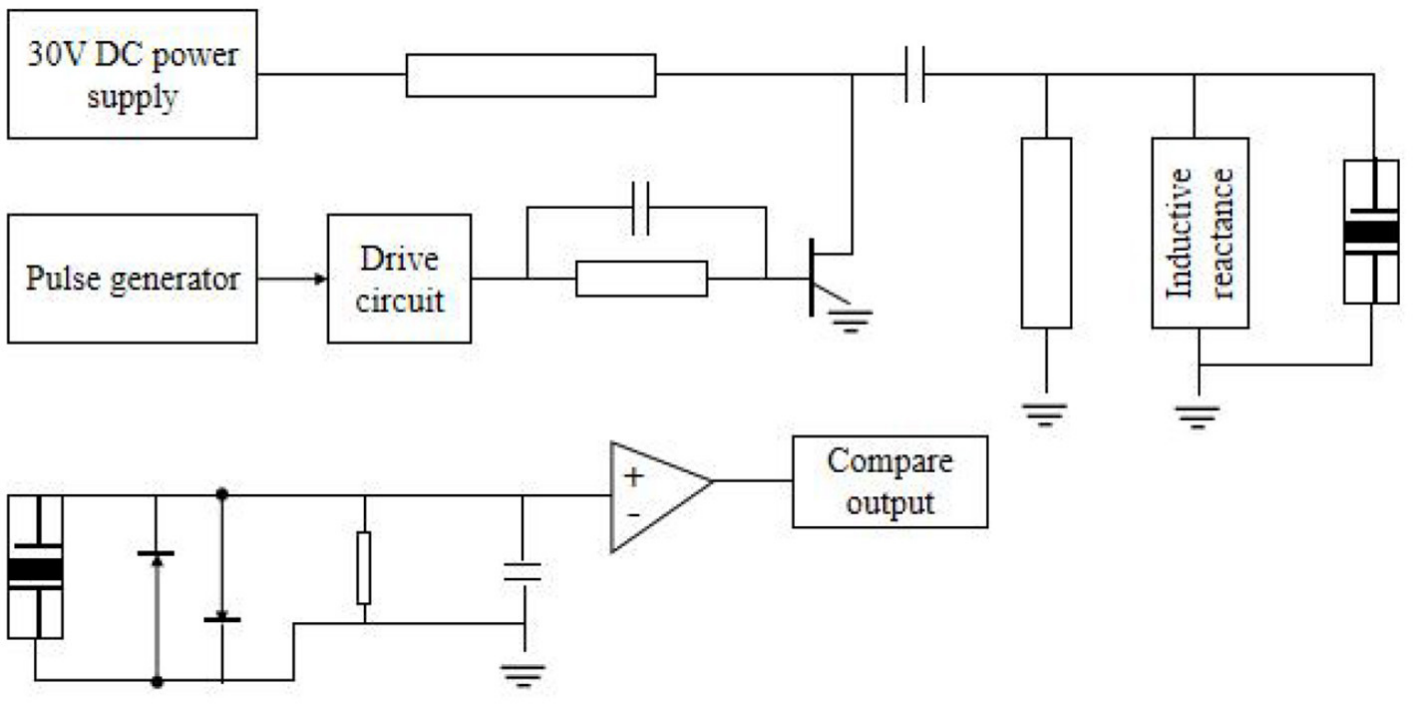
Ultrasonic generator and receiver circuit.

The block diagram of the ultrasonic transmission circuit is shown in Figure 5. The DC stabilized power supply provides energy for the ultrasonic transducer. According to the characteristics of this transducer, it only needs about tens of volts, which can generally be selected within the safe voltage. The current limiting resistance in the circuit is ~1 kΩ/3 W, which is used to limit the ultrasonic energy (Zhenping and Qianhe, 2020). Adjust each limit switch to the edge of the workpiece to be measured so that the moving range of the probe is the range of the workpiece. Because the stimulator converts the rectangular pulse drive into sine wave output through the matching circuit, the ultrasonic intensity control can be realized by changing the driving duty ratio in the ultrasonic cycle. This can not only save the hardware cost but also improve data throughput. The data processing module analyzes and processes the data transmitted from the communication module. The function of the motion control module is to realize interpolation and other control algorithms and control operations.

Extracellular recording signals are electric field change signals generated by the superposition of various transmembrane currents of local neurons. According to the different sizes of signal acquisition electrodes, extracellular recorded signals can reflect the activated state of neurons in different volume ranges in real-time and thus reflect the state of neural circuits stimulated and regulated in real-time.

The generation of brain stimulation signals needs some specific parameters to control, and different stimulation signals need different parameters. The motion control module can control the whole system's motion process according to the detection rules, including setting relative origin, running, resetting, exiting, and other related operations. There is a need to establish and manage channels between network nodes. By means of error control and flow control, the network channel with various interferences becomes more reliable, and the data packets transmitted from the upper layer are converted into bitstreams and sent to the physical layer (Xiaodan et al., 2019). Under the action of capacitor charging and discharging, a negative high voltage pulse wave with the same resonant frequency as the ultrasonic wafer is formed and applied to both ends of the transducer, thereby emitting ultrasonic waves. The inductor in that output circuit is used for eliminating stray capacitance of the cable and capacitive reactance of the ultrasonic transducer. The sensor does not need DC, and the inductor also acts as a bypass.

The microcontroller PIC12F675 generates a pulse with a frequency of approximately 500 kHz, which is output through the I port and supply to the waveform transformation and driving circuit. The pulse frequency here should be consistent with the resonant frequency of the piezoelectric wafer. When bubbles are mixed in the liquid, the amplitude of the detected pulse signal decreases, and the output of the comparator is “low”; otherwise, it is “high.” When the whole liquid level is lower than the mounting plane of the sensor, the output is always “low,” so the sensor can also be used for liquid level monitoring. Any application can easily establish its own SQLite private database according to needs, and by default, other applications cannot directly access the private data file. In the inter-network communication, the high-level protocols of the two processes must be the same, and the situation that one side uses transmission control protocol (TCP) and the other side uses user datagram protocol for data transmission cannot occur.

### Signal Detection and Processing

In the process of using medical equipment, it is generally required to set the monitoring sensitivity according to the actual situation of patients. If the design is too high, it will alarm frequently and fail to work normally, whereas if it is too low, it may lead to malignant accidents. When carrying out ultrasonic testing, the controller should not only make the probe move according to the specified movement track by controlling the operation of the motor but also return the position information of the probe so that the position information can establish a one-to-one correspondence with the ultrasonic digital signals collected by the ultrasonic data acquisition system. The bit-type of the button switch on the touch screen can be converted into the float type required by the serial port server so that the serial port server can receive the correct operation instruction and forward it to the ultrasonic generator; after compression, it is packaged by using relevant protocols, and the header information is added and sent to the mobile device. After unpacking and decoding, the mobile device can display the image information of the ultrasonic device display screen. The static pressure signal corresponding to the point with the largest increasing amplitude is systolic pressure, and the static pressure signal corresponding to the point with the largest decreasing amplitude is the diastolic pressure. Then, the average blood pressure value is calculated by two data of systolic pressure and average pressure.

When the liquid flows normally and no air bubbles are mixed in, the amplitude of the ultrasonic pulse signal detected by the receiving end is large enough, and the pulse signal consistent with the ultrasonic resonance frequency can be obtained by the comparator. Therefore, if the client continuously obtains the screen display information of the server, it needs to adopt a polling mechanism. The corresponding layout file is New patient_edit.xml, which mainly completes the operation of creating new patient information. There are two intents that can jump in. One is when creating a new case, there are no data in the extras part of the intent, and the other is when clicking a piece of information, the intent will carry the data obtained by extras from the database. When there are bubbles passing through the cross-section of the sensor, even when the liquid level is lower than the detection surface of the sensor, the converted value will be greatly reduced. After comparison and digital filtering, an alarm will be generated. By setting the comparison value and the sampling time of A/D conversion, the sensitivity of the alarm can be changed, thus realizing the recognition of bubbles of different sizes.

## Implementation of Medical Ultrasound Remote Control Software

### Implementation of the Measurement Module

In the design of the measurement module, the drawing of primitive is the premise of realizing other more complex functions (Jianqun et al., 2018). To improve the various functions of the software, it is necessary to constantly increase the drawing of various primitives. The medical ultrasound equipment is the server, and the mobile equipment used by users to log in is the client. After the connection between the monitoring terminal and the monitoring software of the upper computer is completed, the five physiological parameters of blood pressure, blood oxygen, electrocardiogram, respiration, and body temperature are collected to debug the collection and transmission functions of each module software. When a device has a communication failure, the serial server will have a communication timeout and try to connect several times, which leads to the high CPU occupancy rate, thus reducing the computing and communication capabilities and affecting normal communication. Because the clock frequency of FPGA is 50 MHz, the single pulse of the stimulation waveform is converted according to the clock frequency, and then according to the adjustment range of various parameters, the binary conversion formula and the corresponding number of bits of parameters can be obtained as shown in Table 1.

**Table 1:** Parameter conversion.

**Parameter**	**Parameter range**	**Transformation relation**	**Digit**
Ft	100 KHz−2 MHz	A = 500/Ft*10	6
PRF	10 Hz−2 KHz	B = 1,000*cpp/Ft*30	22
cpp	0–3,000	C = 110/PRF*8	17
np	0–2,000	D = 110/PRF*8*np	28
SD	0.05–1.5 s	E = 103*SD*6	23
I	0.2–24 W/cm^2^	F = (I + 2.5)/23/7*A	6
Interval	0.3–15 s	G = 103*interval*6	31

In addition, four data bits representing the working state and mode are required, which are output switch, mode selection, continuous or pulse stimulation selection, and single or repeated stimulation selection.

ON_OFF (output switch: output off = 0 output on = 1,);
F_mode (Mode selection: stimulation = 0 positioning = 1,);
S_mode (selection of continuous or pulse stimulation: continuous = 0, pulse = 1,)
T_mode (single or repeated stimulation selection: repeat = 0 single = 1,).

**Figure 6:**
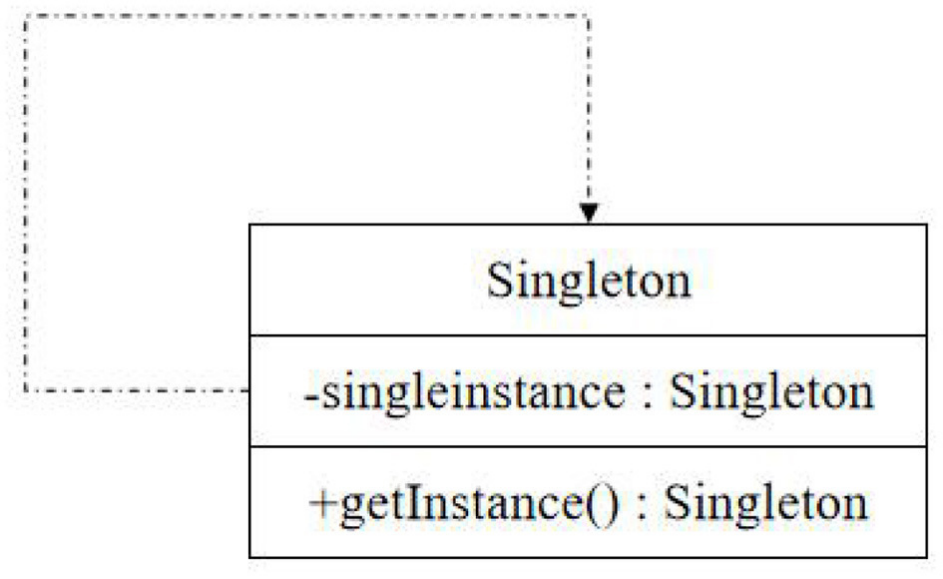
Unified modeling language class diagram of singleton pattern.

Singleton pattern belongs to the creation pattern of objects, which is the simplest design pattern. GOF defines a singleton pattern as follows: ensure that a class can only have one instance object and give a global access point to access the object (Wenchao et al., 2019). The unified modeling language class diagram of the singleton pattern is shown in Figure 6.

File import can be divided into two types: saved echo data and file path: saved echo data are frames of echo data during real-time detection, and a single frame of an echo data file, whose basic parameters, echo defects and echo data, can be directly saved in the database. In addition to creating its own unique instance, the singleton class should also provide a class method with public access rights to access the instance so that it can be accessed in other classes. The file path is when its external line changes; it is difficult to have enough attenuation in the whole speech band because the balance degree of the opposite end changes in different degrees. The measured results show that the opposite end attenuation of the hybrid coil can sometimes be as low as 10 dB at some frequency points. However, the maximum attenuation of the far-end signal after passing through the line is allowed to be 43 dB. Therefore, the echo caused by the end-to-end leakage of the hybrid coil may be 30–40 dB stronger than that of the far-end signal.

Ultrasound equipment will search and match in the database according to the requested username and password. If the authentication is successful, the directory information bound by the user is returned, and the mobile device displays according to the returned directory information. ECG can reflect the activity changes of bioelectricity produced by the human heart in each cardiac cycle. In the debugging of the ECG module, the electrodes of ECG leads are attached to the left and right upper limbs and the left and right lower limbs to measure ECG parameters. By observing the transmission situation, the test shows that within 20 m^2^ around the stimulator, the data transmission is stable, and the Bluetooth connection is effective, which greatly increases the range of activities of the control system.

### Implementation of Network Communication

After realizing the function of monitoring terminal software, it is necessary to realize the data transmission between the monitoring software of the upper computer of the monitoring terminal box through the network. First, search the database to see if the root directory is set. If so, get all the file names in the directory by the list () method and store them in a string group. Then, check the file information, remove the file information that is not a folder, and save only the folder information in the string group. Call helper.get Users () to get qualified information items, then store them in the form of Array List, and add them to Simple Adapter, the object of a simple adapter. Finally, call set Adapter () method to display the found patient information items on the interface. Save it to the database in the form of a binary bitstream. The remote communication module, motion control module, and data processing module of the upper computer Lab VIEW software cooperate with each other to form the upper computer software of the ultrasonic testing multi-axis motion control system.

**Figure 7:**
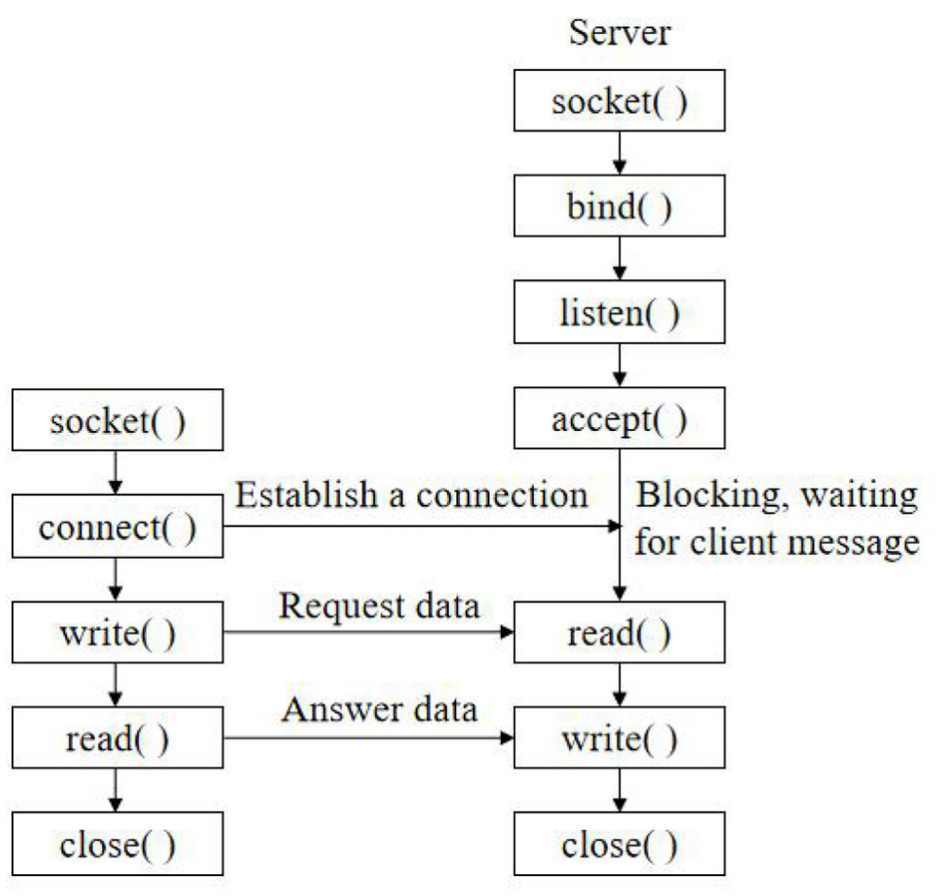
Socket socket programming model.

In this design, the socket socket mechanism is used for network programming to realize data transmission between the monitoring terminal and upper computer monitoring software (Jiatao, 2019). The software is divided into a server part and a client part. The socket socket programming model is shown in Figure 7. In the telemedicine system, the monitoring terminal as the client initiates the communication connection request actively, and the monitoring center as the server is responsible for responding to various connection requests, thus realizing the data communication function between the monitoring terminal and the monitoring center.

Client process and server process use TCP/IP protocol for data communication. First, the client initiates a connection establishment request to the server, and then, the server accepts the client's connection request. Patients and medical staff can log in to the account on the mobile device client to obtain the file directory information on the ultrasonic device and preview, download, print, and other operations, which greatly facilitates doctors and patients to use medical records. When a communication failure occurs between a certain device and the serial server, the system automatically extends the communication cycle of the failed device and then restores the communication cycle to the normal value after a certain communication is normal. The Ethernet communication protocol between the multi-axis motion controller and the upper computer Lab VIEW is defined to realize the remote communication between the lower computer and the upper computer. Add the string group to the adapter, and add special strings to the adapter, so that the user can click this option to return to the higher level directory, and bind the adapter with List View, so that List View can display these string information.

The client–server model describes the relationship between services and serviced processes, in which the client is the service requester, and the server is the service provider. Client/server (C/S) mode has strong interactivity, good real-time and flexibility, and strong data manipulation and transaction processing capabilities. The specificity and closeness of the adopted protocol make the whole C/S mode system relatively safe, so this system adopts TCP/IP protocol and is based on C/S operation mode. This way implies the inequality of hardware resources between client/server and the asymmetry of communication. In actual measurement and control, multiple photoelectric measurement and control devices measure at different stations at the same time, and the data processing center coordinates and monitors multiple measurement stations (Zhuo, 2019), that is, the structure of one client to multiple servers (the client is the data processing center). Clinicians and patients need to register their own accounts and set their own usernames, login passwords, and corresponding directory files before applying the remote file management module.

### Implementation of Screen Sharing Function

Due to the limitation of system level, the traditional method of obtaining screen information can only obtain screen data by calling frame buffer through JNI, which is the main reason for the poor quality of screen sharing. If you want to delete, just let the patient information item to be deleted get the focus first and then click Delete Medical Record Item. If you want to re-edit the existing information, you only need to click the corresponding patient information item to enter the editing interface, save it after modification, and then return to the patient information management interface. After the client request comes, accept the connection request through the accept () function and return a new socket corresponding to this connection. Use the returned socket to communicate data with the client and perform read and write operations. After data transmission, the socket waits for another request from the client. Therefore, the CPU of the system is maintained at a low level, and the communication with other devices will not be affected after a single communication failure.

**Figure 8:**
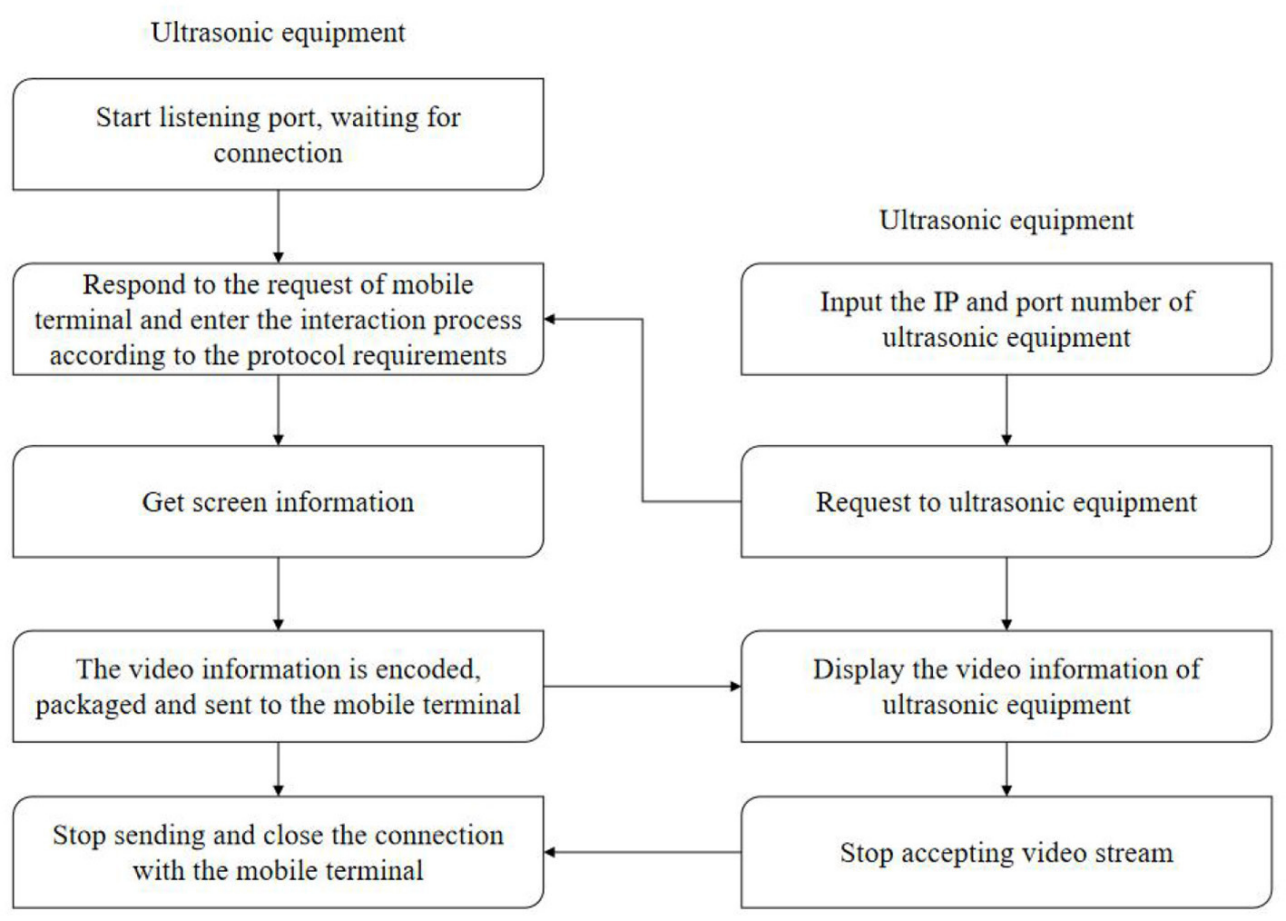
Screen sharing implementation flowchart.

In terms of transmission mode, the conventional unicast protocol is mainly used for point-to-point transmission, and multicast protocol is used for transmission in this subject. The flowchart of screen sharing is shown in Figure 8.

There may be different types of files in the memory card. If you open it for viewing, you need to start the corresponding activity. You can first get the path and file name of the file and determine a uniform resource identifier. H.264 encoded video stream can adapt to bandwidth and can generate video stream suitable for network transmission according to needs (Yanping et al., 2018); The FPGA controls the A/D conversion circuit to convert the ultrasonic echo signal and stores the data in the dual-port random-access memory. After the storage is completed, it sends a signal to ARM, and ARM receives the acquisition completion signal and sends the data to the upper computer through Ethernet. Reset is to return the probe to the absolute zero position. Origin setting can set the current arbitrary scene as the relative origin, as the starting point of the new movement and as the relative zero point of the probe position coordinates. After the connection is successful, the program enters the cycle of sending and receiving data until the connection is closed. If the reception is successful, the data will be processed accordingly; if it is unsuccessful, the close () function will be called to close the current connection.

After the medical ultrasound equipment software is started, the screen real-time sharing service is started, and the response port is monitored. When a mobile device is connected, a request will be sent to the corresponding port of the medical ultrasound equipment, and the medical ultrasound equipment will respond to the request of the mobile device and start to acquire screen information. For the sake of holding the medical ultrasound device, the operation should be done on the touch screen as much as possible. Click a frame of image lightly, and the image display area will be displayed accordingly. The body temperature of the human body is measured by the upper computer monitoring software to verify the monitoring performance of the upper computer monitoring software on body temperature. The data can be stored in an Excel file, the data file of each test can be opened and called, the relevant information can be read, and the test image can be generated by combining with image processing software. According to the video streaming protocol and the compression protocol, the obtained video information is displayed on the screen of the mobile device.

## Conclusion

In this paper, the application programs of mobile devices and medical ultrasound devices are developed based on sensor design so that users can use mobile devices to remotely control medical ultrasound devices in the same LAN, view shared screens, and manage file systems and other remote operations. According to the communication protocol between monitoring software and host computer monitoring software, the development platform of host computer monitoring software is built, and the design and implementation of host computer software are completed. The network communication between the monitoring terminal and the monitoring software of the upper computer is realized by socket programming under TCP/IP. The upper computer software of ultrasonic testing multi-axis motion control system based on Ethernet is written by Lab VIEW, which realizes the processing of ultrasonic testing control signals by the upper computer and the automation of ultrasonic testing remote control. It realizes not only the local automatic control of the ultrasonic vibration system but also the remote control of the operator station, which greatly improves the automation level of the ultrasonic system.

This design has basically achieved the expected design effect, but there are still some deficiencies in system stability, practicability, scalability, and network communication, which need to be improved and perfected continuously. In the next step, we can expand the use range of telemedicine from LAN to wide area network so that the coverage of LAN is no longer the main limitation.

## Data Availability Statement

The original contributions presented in the study are included in the article/supplementary material, further inquiries can be directed to the corresponding author/s.

## Author Contributions

QJ and YZ participated in the design and coordination of experimental work, and acquisition of data. YZ carried out the study design, the analysis and interpretation of data, and drafted the manuscript. All authors read and approved the final manuscript.

## Conflict of Interest

The authors declare that the research was conducted in the absence of any commercial or financial relationships that could be construed as a potential conflict of interest.
